# Guided Implant Surgery to Reduce Morbidity in Von Willebrand Disease Patients: A Case Report

**DOI:** 10.2174/1874210601812010080

**Published:** 2018-01-31

**Authors:** Mathilde Fénelon, Sabine Castet, Jean-Christophe Fricain, Sylvain Catros

**Affiliations:** 1 Department of Dentistry and Oral Health, University Hospital of Bordeaux, Bordeaux, France; 2 Inserm U1026, BioIngénierie Tissulaire, University of Bordeaux, Bordeaux, France; 3 Center for Inherited Bleeding Disorders (CRTH), University Hospital of Bordeaux, Bordeaux, France

**Keywords:** Dental implant, Oral surgery, Von Willebrand disease, Bleeding disorder, Hemostasis, Guided surgery

## Abstract

**Introduction:**

Von Willebrand Disease is the most common inherited bleeding disorder. In the general population, 1/8000 patients are affected. Primary hemostasis (platelet adhesion) and coagulation (protection of Factor VIII) are altered. Among several bleeding symptoms, these patients suffer from excessive bleeding of oral mucosa and dental management requires a close collaboration between haematologists and oral surgeons.

**Materials & Methods:**

Guided implant surgery can be used to increase the accuracy of implant placement and to reduce the overall morbidity of this surgical procedure by using a flapless surgery technique.

**Case Report:**

We report the case of a 49 years old woman having a Type 2A von Willebrand disease and who presented to replace tooth #.46 because of interradicular fracture and peri-apical infection. After planning the implant surgery using Codiagnostix^®^ software, a surgical guide was prepared. The patient received 4 injections of von Willebrand factor (Willfactin^®^) for this particular surgical procedure. The implant was placed immediately after tooth removal and local haemostasis was performed.

**Discussion:**

The follow-up was uneventful and the implant was restored by a crown 4 months later. Two cases of implant placement in haemophiliac patients have been reported before in the literature.

**Conclusion:**

As far as we know, this is the first case report of implant placement in a patient having a von Willebrand disease. The use of guided surgery allowed to perform a mini-invasive procedure and thus contributed to prevent bleeding complications in this patient.

## 1 INTRODUCTION

Von Willebrand Disease (vWD) is the most common inherited bleeding disorder, due to a quantitative or a qualitative defect of von Willebrand Factor (vWF), and follows an autosomal inheritance pattern [1]. In the general population, depending to diagnostic criteria, vWD prevalence varies between 1/100 to 1/10000. The vWF gene is located on the short arm of chromosome 12. Both primary hemostasis and coagulation are altered. In primary hemostasis, vWF facilitates platelet adhesion to the sites of vascular injury through glycoprotein Ib (GPIb) receptor. Besides, to achieve secondary hemostasis, vWF stabilizes and protects factor VIII [2, 3]. Hereditary vWD are classified into three types: Type 1, Type 2, and Type 3 according to the qualitative or quantitative deficiency of vWF. Type 1 (Frequency: 70% - 80%) results from a quantitative deficiency of a functionally normal vWF. Type 2 (Frequency: 20%) is characterized by a qualitative defect in VWF, resulting in a dysfunctional protein. Different subtypes of Type 2 vWD have been defined (2A, 2B, 2N, 2M). Type 3 (Frequency <5%) results from an almost complete deficiency of vWF [1, 3].

The clinical diagnosis of vWD can be evoked on the personal history of bleeding (mucocutaneous bleeding, menorrhagia, epistaxis, gastrointestinal bleeding, previous postsurgical bleeding), a family history of bleeding, or both. Then it is confirmed with systematic laboratory tests revealing abnormalities in vWF, factor VIII, or both [1, 2]. The most common symptoms in patients with vWD are bruising, hematomas, epistaxis, menorrhagia, and extended bleeding after minor wounds [1].

Among these numerous bleeding symptoms, these patients suffer from frequent bleedings located in the oral mucosa and dental management requires a close collaboration between hematologists and oral surgeons [2,4-6]. The amount and severity of bleedings depend on the type of the vWD, both local and systemic patient factors (such as periodontal inflammation, vasculopathy or platelet dysfunction) and surgical-related factors.

A recent review, which evaluated dental implant treatment in the medically compromised patient concluded that there is no evidence that any bleeding disorders are an absolute contraindication to dental implant surgery, although these patients may be at risk of prolonged hemorrhage and blood loss [7].

The present report describes the management of a type 2 vWD patient, who underwent a dental implant surgery, immediately after tooth extraction.

## 2 CASE REPORT

A 49 years old woman suffering from Type 2A von Willebrand disease, presented in the dentistry and oral health department, University Hospital of Bordeaux (Bordeaux, France). The last results of the biological exploration regarding coagulation revealed the following parameters: F VIII = 16%; vWF RCo = 7%; vWF Rag = 12%.

**Fig. (1):**
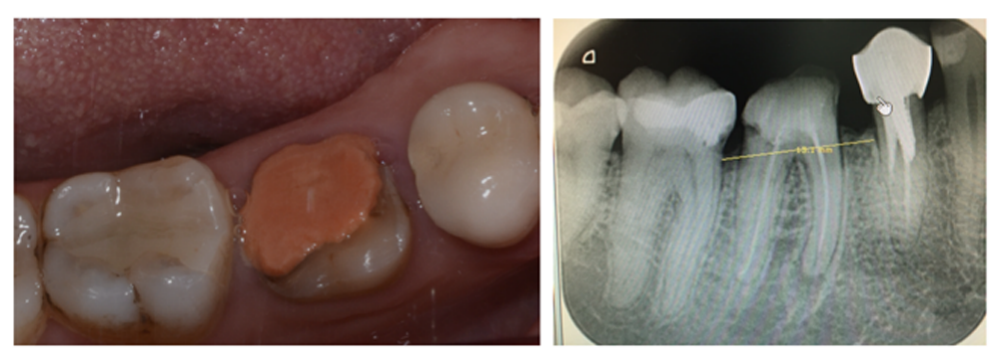
Initial situation A) Pro-operative clinical view. (B) Preoperative intraoral radiograph of tooth 46.

**Fig. (2):**
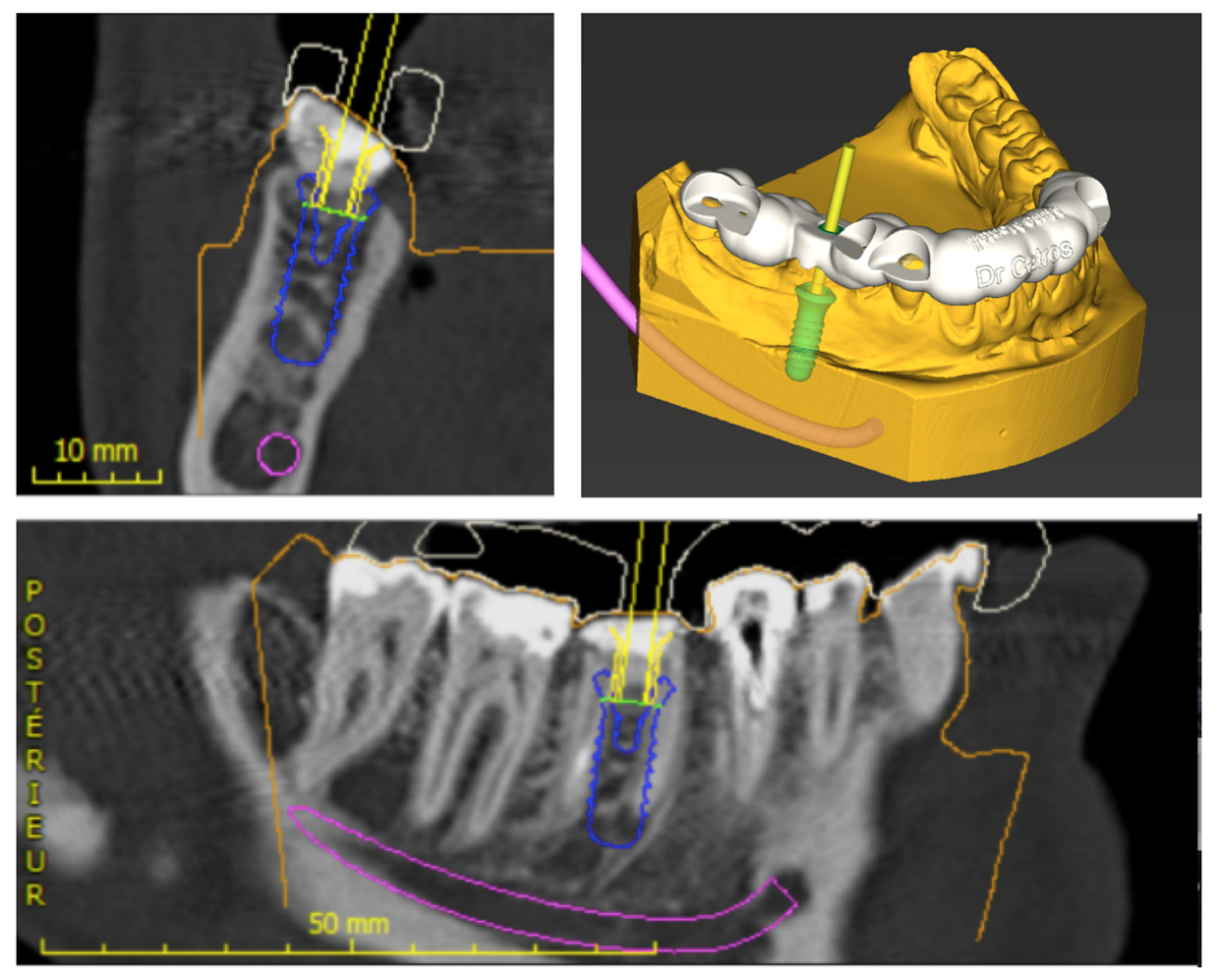
Computing 3D planning for implant position. (A) Cross-sectional image of virtually placed implant. (B) Three-dimensional image of the surgical guide. (C) The implant planning.

She was referred by her general dentist to remove tooth #46 and to replace it by a dental implant. The lower first molar had to be removed because of inter radicular fracture and peri-apical infection (Fig. **1**). A Cone-Beam Computed Tomography (CBCT) and an optical impression were recorded. Codiagnostix^®^ software was used to match DICOM and STL data. It revealed that sufficient remaining alveolar bone support was present in the interradicular space of 46 to stabilize an implant immediately after tooth extraction (Fig. **2A**). This was proposed to the patient and she gave her consent for this surgical procedure. A surgical guide was 3D printed using Codiagnostix^®^ planification (Fig. **2B**).

**Fig. (3):**
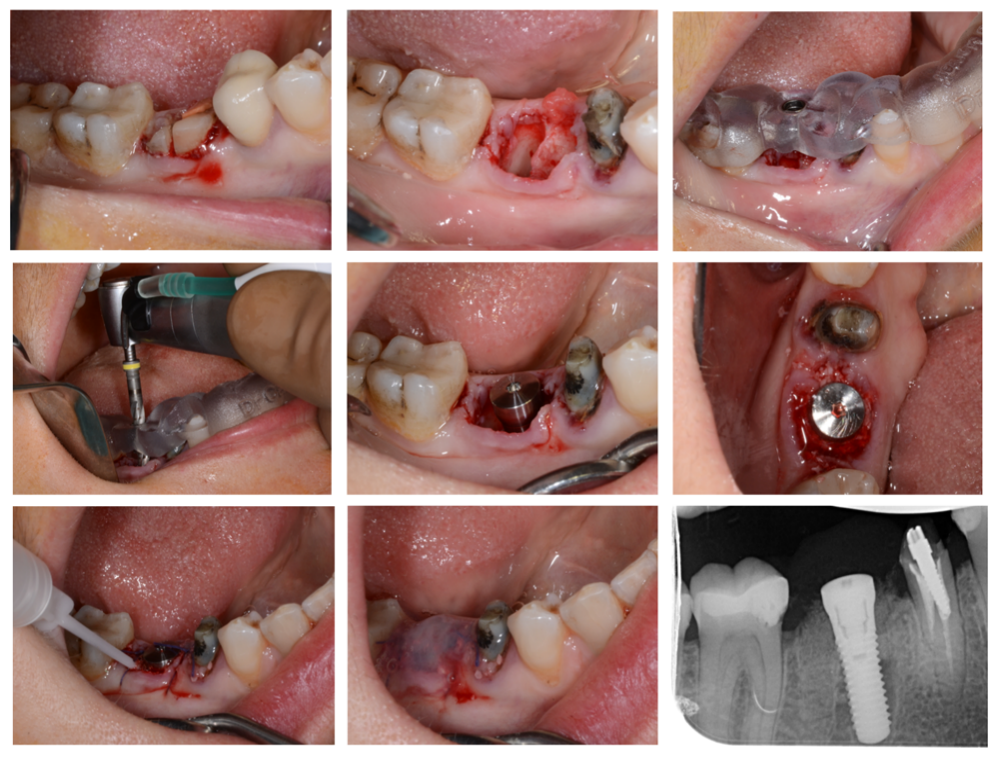
Surgery (A,B,C) Flapless extraction of the tooth after root hemisection to preserve the septal bone. (D,E,F) Guided implant surgery. (G,H) Fibrin glue was applied on the surgical wound all around the healing abutment. (I) Post-operative intraoral radiograph.

The patient was hospitalized the day of surgery and received one injection of von Willebrand factor (Willfactin^®^, 2500 UI) one hour before the surgical procedure. It was performed under local anesthesia (Primacaine^®^ supplemented with 1/200 000 Adrenalin v/v). No flap was raised and the tooth was carefully extracted after root hemisection (Fig. **3A**). Curettage and alveolar debridement were performed. Then, the surgical guide was stabilized to the adjacent teeth so the implant could be placed immediately after tooth removal. Straumann^®^ Guided Surgery was used, following the surgical sequence determined during the planification. A torque of 35 N was obtained at implant placement (Straumann^®^ TE 4.1x12mm RN) and the healing abutment (3 mm) was immediately placed on the implant (Fig. **3B**). A xenograft bone-substitute material (Bio-Oss^®^ Small granules 0,25-1mm) was used to fill the bone defects between the alveolar socket and the implant.

Local hemostasis was performed with absorbable sutures (VICRYL^TM^ 4.0 ETHICON^®^) and fibrin glue (Tisseel^**®**^, 2ml, Baxter AG) was applied on the surgical wound (Fig. **3C**). No excessive bleeding was observed during or after the surgical procedure. The patient received one injection every twelve hours of von Willebrand factor (Willfactin^®^, 2500UI) during 48 hours, before discharge. Post-operative prescriptions included *per os* Tranexamic acid (EXACYL^®^ 1g/10ml, 3 times per day during ten days), Paracetamol (1g, 3 times per day during 4 days) and Amoxicilline (1g, twice a day, during seven days). The follow-up was uneventful and the implant was restored by a crown 4 months later.

**Fig. (4):**
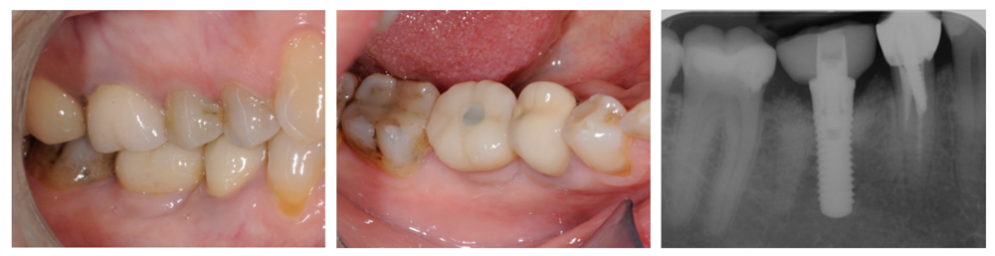
Clinical (A,B) and radiographic (C) view on year after implant placement.

1 year after final restoration, clinical and peri-apical x-rays status were normal (Fig. **4**).

## 3 DISCUSSION

Spontaneous or post-traumatic bleeding can occur in the oral cavity of vWD patients, due to the alteration of both primary hemostasis and coagulation [1, 3]. This increased risk of postoperative bleeding after a surgical procedure, justifies a close collaboration between hematologists and oral surgeons before planning electives surgical interventions. Thus, a specific hemostatic protocol can be determined for each patient and each procedure [2, 4].

Therapeutic agents to prevent or control bleeding in vWD patients are classified into three categories. First, in the less severe bleeding phenotypes, Desmopressin (DDAVP) can be administered I.V. or by intra-nasal spray to increase the plasma concentration of vWF by releasing endogenous vWF stocks through stimulation of endothelial cells. Then, in more severe cases, a substitution of the vWF can be used by I.V. injection. Finally, therapies enhancing alternative prothrombotic pathways or decreasing fibrinolysis, without substantially altering the plasma concentration of vWF might be used in some specific situations [2, 4]. These treatments could be administered together or separately depending on the type and severity of vWD [2].

Desmopressin (DDAVP) increases plasma vWF levels but it is usually ineffective in patients with Type 2A disease. That’s why our patient received a replacement therapy, which consisted of one injection of vWF (Willfactin^®^, 2500 UI) before the surgical procedure and one injection every twelve hours until her discharge.

One study recommended administration of 50-90 U. kg^-1^. as ristocetin cofactor (VWF:RCof) of factor VIII concentrate containing von Willebrand factor (FVIII/VWF concentrate) for patients with type 2A VWD before dental extractions. This should be administered twice in routine extractions, and four to six times in surgical extractions, associated with the use of tranexamic acid [6].

Two other therapies to promote hemostasis were used in this case. First, a topical fibrin glue was applied on the surgical wound to enhance local hemostasis. Then, tranexamic acid was administered orally to the patient during ten days after the surgical procedure. Fibrin glue mimics the final stage of the coagulation. Similar to local haemostatic measures and suturing, antifibrinolytic therapy is a safe and cost-effective treatment with beneficial effect in preventing postoperative bleeding in individuals with bleeding disorders undergoing oral surgery. However, to date, there is not enough evidence to conclude definite efficacy of antifibrinolytic therapy in oral or dental procedures in patients with inherited bleeding disorder such as haemophilia or vWD [8].

Severe bleeding complications are rare in dental implant surgery. The most frequently reported cause of serious postoperative bleeding in dental implant surgery is related to the perforation of the lingual plate of the mandible, especially when the implants are placed in the mandibular symphysis and lead to a damage of the sublingual artery [9]. Even though mild haemorrhage can be a relatively common complication in dental implant surgery, there is no reliable evidence to suggest that bleeding disorders are a contraindication to the placement of implants, although these patients may be at risk of prolonged bleeding after surgery [7].

Two cases of implant placement in haemophiliac patients have been reported before in the literature [5, 10]. One study reported an implantation procedure due to traumatic loss of the teeth 21 and 22 in a patient suffering from a mild form of haemophilia A. The replacement therapy consisted in the administration of factor VIII (Beriate^®^, Behring, Marburg, Germany, 3×1.000 IU), and no adverse events were noted [5]. No follow-up data were reported concerning osseointegration or prosthesis after implant placement. Another study described the surgical and restorative procedures in a patient suffering from moderate haemophilia A [10]. Eight teeth had to be removed before five implants could be placed to restore the edentulous space. Four separate surgical procedures were performed and all surgical procedures were performed on an outpatient basis. Before each surgical procedure, the patient received 2.500 UI of factor VIII 1 hour before surgery and 1.000 UI 12h after surgery. Tranexamic acid (2.5 g, 8h before surgery and again every 8h for ten days) was also administered. This preparatory regimen of treatments, along with local hemostasis, controlled effectively intraoperative and postoperative bleeding. Hemostasis was accomplished so that osseointegration could proceed normally and a final restoration was performed with a cement retained, implant-supported prosthesis [10].

A specific surgical procedure was used to reduce the morbidity of the procedure and to limit potential bleeding complications in our patient. First, the remaining tooth was extracted carefully after root separation and no flap was raised. Inflammatory and infected tissues were carefully removed from the sockets. The surgical planification was done using a dedicated software to evaluate the feasibility of the procedure. It revealed that the implant could be placed safely in the interradicular septum, because the remaining molar possessed thin and divergent roots, which represent the most favorable situation for this type of surgical procedure [11].

Guided Surgery finally allowed to perform a flapless surgery and the surgical time was reduced, both conditions being highly favorable to reduce post-operative bleeding. It was shown before that using flapless guided surgery provides significantly less immediate post-operative hemorrhage than open-flap guided surgery and conventional open flap surgery [12]. The final implant position was similar to the planification thus allowing to perform a conventional screw-retained prosthetic restoration [13].

Local hemostatic techniques are fundamental to manage oral surgery the patients displaying inherited bleeding disorders. In this case, an intra-alveolar compression was done first by the implant itself, then by the particulate biomaterial packed around the remaining bone defects in the sockets. The role of the biomaterial granules was also to favor bone formation in bone defects >2mm [14]. Finally, sutures and fibrin glue were used to complete the compression and to maintain the blood clot in the first hours after the surgical procedure [2]. The oral administration of tranexamic acid was used to delay the early disintegration of the blood clot [8].

## CONCLUSION

As far as we know, this is the first case report of implant placement in a patient having a von willebrand disease. Use of guided surgery allowed (i) to perform a single mini-invasive procedure to reduce the risk of potential bleeding complications, (ii) to reduce the total treatment duration and (iii) to avoid recurrent injections of vWF, which are highly expensive.

## LIST OF ABBREVIATIONS

vWD: von Willebrand Disease
vWF: von Willebrand Factor
